# Comparison of Efficacy and Safety of Magnetic Seizure Therapy and Electroconvulsive Therapy for Depression: A Systematic Review

**DOI:** 10.3390/jpm13030449

**Published:** 2023-02-28

**Authors:** Dong-Bin Cai, Xin-Hu Yang, Zhan-Ming Shi, Sha Nie, Rui Xu, Xiu-De Qin, Xiong Huang, Xing-Bing Huang, Wei Zheng

**Affiliations:** 1 Shenzhen Traditional Chinese Medicine Hospital, Shenzhen 518033, China; 2 The Affiliated Brain Hospital of Guangzhou Medical University, Guangzhou 510260, China; 3 Chongqing Jiangbei Mental Health Center, Chongqing 510000, China

**Keywords:** magnetic seizure therapy, electroconvulsive therapy, depression, systematic review, efficacy

## Abstract

Objectives: As a new physical therapeutic technique, magnetic seizure therapy (MST) has established efficacy in the treatment of depression with few cognitive side effects, and thus appears to be a potential alternative to electroconvulsive therapy (ECT). The findings of randomized controlled trials (RCTs) examining the efficacy and safety of MST versus ECT for depression are inconsistent. This systematic review of RCTs was designed with the aim of assessing the safety and efficacy of MST versus ECT for patients with depression. Methods: The WanFang, Chinese Journal Net (CNKI), EMBASE, PubMed, Cochrane Library, and PsycINFO databases were systematically searched by three independent investigators, from their inceptions to July 24, 2021. Results: In total, four RCTs (n = 86) were included and analyzed. Meta-analyses of study-defined response (risk ratio (RR) = 1.36; 95% CI = 0.78 to 2.36; *p* = 0.28; I^2^ = 0%), study-defined remission (RR = 1.17; 95% CI = 0.61 to 2.23; *p* = 0.64; I^2^ = 0%), and the improvement in depressive symptoms (standardized mean difference (SMD) = 0.21; 95% CI = −0.29 to 0.71; *p* = 0.42; I^2^ = 0%) did not present significant differences between MST and ECT. Three RCTs evaluated the cognitive effects of MST compared with ECT using different cognitive measuring tools, but with mixed findings. Only two RCTs reported adverse drug reactions (ADRs), but these lacked specific data. Only one RCT reported discontinuation due to any reason. Conclusions: This preliminary study suggests that MST appears to have a similar antidepressant effect as ECT for depression, but mixed findings on adverse cognitive effects were reported.

## 1. Introduction

Depression is a severe mental disorder with a high suicide rate and heavy disease burden, with a large impact on individuals and on society [1,2,3]. Patients with depression first used antidepressant therapies combined with psychotherapy, but approximately 50% of patients with depression still failed to respond to standard treatments, and approximately 33% of patients with depression had not responded to at least two different antidepressants, or medication combined with psychotherapy [4,5]. To date, electroconvulsive therapy (ECT) is regarded as a highly effective therapy for patients with major depressive episodes, where remission is greater than 60%, according to clinical practice and clinical guidelines [6,7,8]. Nevertheless, ECT is frequently associated with cognitive side effects, limiting its widespread use [9,10,11].

Magnetic seizure therapy (MST) is a new physical therapeutic technique that uses alternating strong magnetic fields to perform generalized seizures for antidepressant-therapeutic purposes [12,13,14]. Numerous studies have proved that MST has similar antidepressant efficacy to ECT and few adverse effects in treating major depressive disorder (MDD) [15,16,17,18,19]. Similarly, a recent systematic review reported that MST, as an adjunctive treatment, was efficacious for total psychopathology in schizophrenia [20]. Relative to transcranial magnetic stimulation (TMS), a larger output voltage and higher magnetic field frequency are administered using MST, constituting a more effective method for treating psychiatric disorders [21,22,23].

Several previous studies have proved that MST can change the metabolism of the bilateral frontal cortex of patients with depression, which could improve depression symptoms without adverse cognitive deficits [24,25]. For example, two case reports found that two MDD patients reported a superior improvement in their depressive symptoms after MST treatment [26,27]. Several observational studies have shown that MST results in a clinically meaningful improvement in depressive symptoms in MDD patients and produces negligible cognitive impairment [24,25,28,29]. Notably, a recent meta-analysis, including randomized controlled trials (RCTs) (n = 4) [15,17,30,31] and non-RCTs (n = 6) [16,32,33,34,35,36], found that MST leads to shorter recovery and reorientation times for individuals suffering from depression than ECT [19]. However, four RCTs [15,16,17,18] have compared the antidepressant effects and safety of MST and ECT for depression, but with mixed findings.

Two recent systematic reviews and meta-analyses [19,37] were conducted to compare the antidepressant and cognitive effects of MST and ECT, finding that the antidepressant effects of MST are equivalent to those of ECT. The common limitations of these meta-analyses included one study [30] with inconsistent reports about whether the study samples were randomly assigned when compared with their study protocol (NCT01318018). Furthermore, another study [38], using the same databases as Kayser et al.’s study [30], pointed out that the random allocation of patients to the ECT and MST groups was impossible, due to both institutional and organizational reasons. Another meta-analysis [19] included 10 studies (n = 285) [15,16,17,30,31,32,33,34,35,36], but 6 out of 10 (60%) were non-RCTs [16,32,33,34,35,36], the inclusion of which violated standard recommendations. Thus, we conducted this systematic review including an additional RCT [18] to compare the antidepressant effects and adverse cognitive functions of MST and ECT for depression.

## 2. Methods

This systematic review was performed in line with the Preferred Reporting Items for Systematic Reviews and Meta-Analyses (PRISMA) guidelines [39].

### 2.1. Inclusion Criteria

The inclusion criteria were described according to the following ***PICOS*** acronym. Participants: The patients were aged at least 18 years old and were diagnosed with MDD or bipolar depression (BD) according to any international diagnostic criteria. Intervention and Comparison: We considered MST plus treatment as usual (TAU) versus ECT plus TAU. Outcomes: The primary outcomes were study-defined response (e.g., ≥50% reduction in Hamilton Depression Scale (HAMD) total scores) and study-defined remission (e.g., HAMD total scores ≤ 7 or Montgomery–Äsberg Depression Scale (MADRS) total scores ≤ 10) at endpoint assessment and follow-up assessment. Key secondary outcomes: the improvement in depressive symptoms (e.g., HAMD total scores or MADRS total scores), cognitive functions, adverse drug reactions (ADRs), and any reason for discontinuation. Study design: Only RCTs evaluating the efficacy and safety of MST versus ECT were included. Case reports, observational studies, non-RCTs, reviews, and meta-analyses were excluded.

### 2.2. Study Selection

Three investigators (DBC, XHY, and ZMS) individually searched the six major bibliographic databases (Chinese Journal Net, WanFang, EMBASE, PubMed, Cochrane Library, and PsycINFO) from their inceptions to 24 July 2021. The keywords used in the search were as follows: (magnetic seizure therapy [MeSH Terms] OR magnetic seizure therapy OR MST) AND (electroconvulsive therapy [MeSH Terms] OR electroconvulsive therapy OR ECT) AND (depression [MeSH Terms] OR depression OR depressive OR depressed OR melancholia). Furthermore, three investigators (DBC, XHY, and ZMS) independently hand-searched the references of the included RCTs [15,16,17,18], related review [14], and meta-analyses [19,37] to obtain missing RCTs.

### 2.3. Data Extraction

Three independent investigators (DBC, XHY, and ZMS) extracted the data from each included RCT using a standard Microsoft Excel (Microsoft office 2016, Redmond and United States) file. Inconsistencies were resolved through consensus involving a senior investigator (WZ). Furthermore, missing data for each included RCT were obtained by sending emails to the first author and/or the corresponding author, if necessary.

### 2.4. Quality Assessment

The Jadad scale [40] and Cochrane risk of bias tool [41] were applied by three independent investigators (DBC, XHY, and ZMS) to examine the quality of each included RCT. The Grading of Recommendations Assessment, Development, and Evaluation (GRADE) system was applied by three independent investigators (DBC, XHY, and ZMS) to determine the overall evidence quality level of all pooled results from the included RCTs [42,43].

### 2.5. Statistical Analyses

Data were pooled by applying the random-effect model [44] and Review Manager software (version 5.3, London and United Kingdom). Standardized mean differences (SMDs), with 95% confidence intervals (CIs) for continuous data, and risk ratios (RRs), with 95% confidence intervals (CIs) for dichotomous data, were computed. Heterogeneity is expressed with I^2^ and *p* values. A *p* value < 0.1 or I^2^ ≥ 50% suggests significant heterogeneity. To detect the reasons for the significant heterogeneity, subgroup or sensitivity analyses were performed if necessary. In this systematic review, Egger’s test [45] and funnel plots [46] were implemented to identify publication biases. All meta-analyses were 2-tailed, with alpha = 0.05.

## 3. Results

### 3.1. Results of the Search

**Figure 1:**
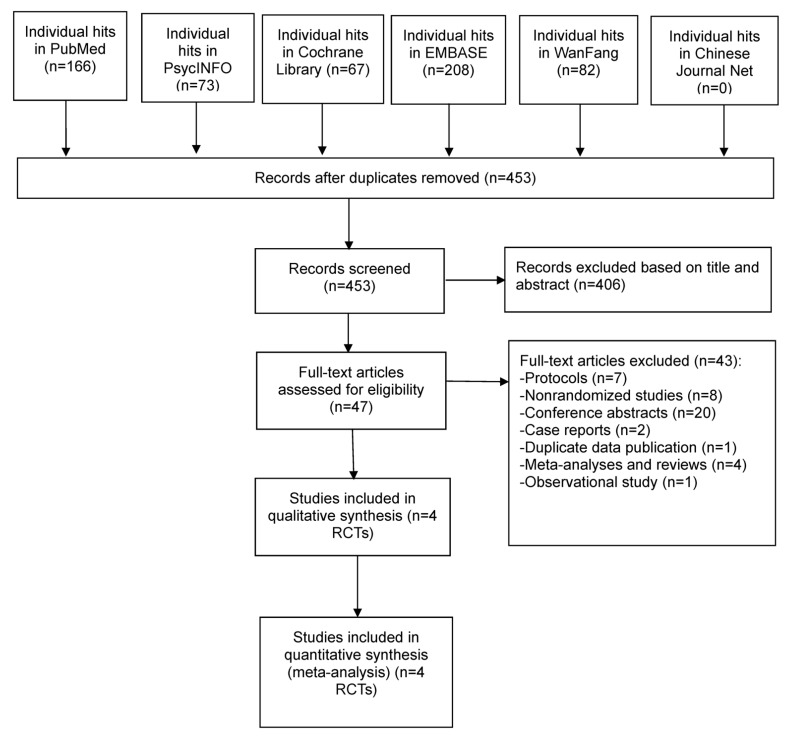
PRISMA flow diagram.

As reported in Figure 1, a total of 596 articles were initially identified in the above 6 databases, 143 duplicates were removed and 47 articles were full-text screened. One study [30] and its study protocol (NCT01318018) had inconsistent reports about whether the study samples were randomly assigned and whether they were excluded. Finally, 4 RCTs [15,16,17,18] involving 86 patients fulfilled the inclusion and exclusion criteria of this systematic review.

### 3.2. Participants and Study Characteristics

The participants and study characteristics are presented in Table 1. Four RCTs [15,16,17,18] compared the MST group (n = 44) with the ECT group (n = 42). The sample sizes ranged from 6 to 40. The weighted mean age was 49.1 years old (range from 45.9 to 64.8 years) in four RCTs [15,16,17,18], and the mean duration of illness ranged from 3.6 to 25.2 years (the weighted mean illness duration = 49.1 years) in three RCTs [15,16,17]. The study duration ranged from 3 to 6 weeks, and the number of either ECT or MST sessions ranged from 8 to 18 across all of the included RCTs [15,16,17,18]. Among the four RCTs, the electrode placement of MST varied, including bilaterally at F5–F6 of the international 10–20 electroencephalograph (EEG) system.

### 3.3. Quality Assessment and Publication Bias

As depicted in Supplemental Figure S1, only one RCT (25%, 1/4) reported randomization methods with a detailed description [15], while three RCTs (75%, 3/4) were valued as low risk with regard to attrition bias [16,17,18]. The weighted mean Jadad score was 2.5 (range from 1 to 5) (Table 1), and one RCT (25%, 1/4) was considered high quality (Jadad score = 5). The quality of evidence of all meta-analyzable results in this systematic review was valued as “moderate” (100%, 3/3) using the GRADE approach (Table 2).

Publication bias for primary and secondary outcomes could not be determined in this study using Egger’s test or a funnel plot (<10 trials) [46].

### 3.4. Antidepressant Outcomes

**Figure 2:**
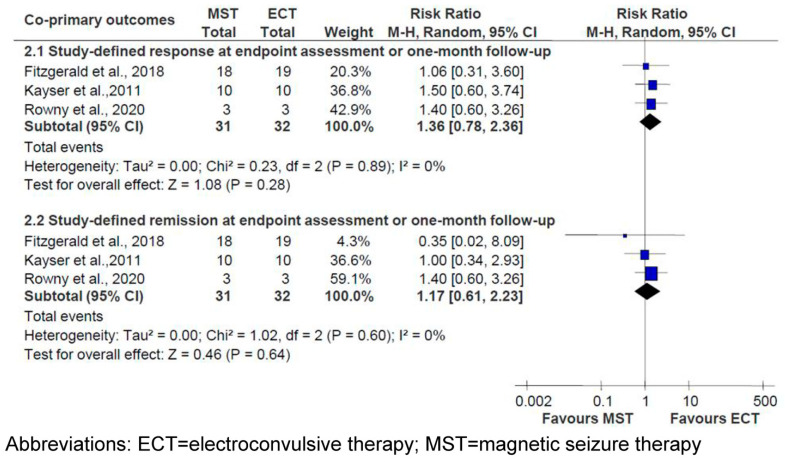
MST versus ECT for depression: forest plot for study-design response and remission [15,16,18].

**Figure 3:**
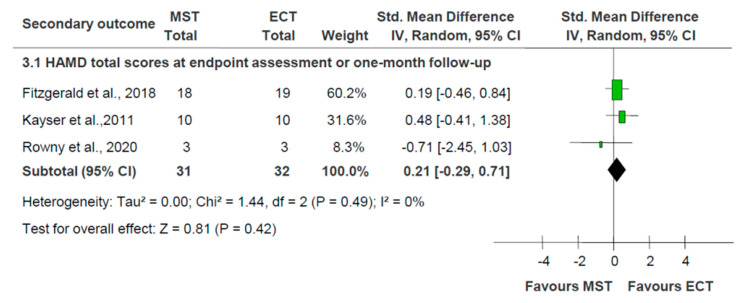
MST versus ECT for depression: forest plot for the improvement of depressive symptoms as measured by the HAMD. Abbreviations: ECT = electroconvulsive therapy; HAMD = Hamilton Depression Scale; MST = magnetic seizure therapy [15,16,18].

Meta-analyses of study-defined response (three RCTs: n = 63; RR = 1.36; 95% CI = 0.78 to 2.36; *p* = 0.28; I^2^ = 0%), study-defined remission (three RCTs: n = 63; RR = 1.17; 95% CI = 0.61 to 2.23; *p* = 0.64; I^2^ = 0%) and HAMD total scores (three RCTs: n = 63; SMD = 0.21; 95% CI = −0.29 to 0.71; *p* = 0.42; I^2^ = 0%) at the endpoint or one-month follow-up assessment period did not find a significant effect of MST versus ECT [15,16,18] (Figure 2 and Figure 3). The antidepressant effects of MST and ECT, as measured by other standardized rating scales, such as the MADRS, were also similar [15,16] (Supplemental Table S1).

### 3.5. Cognitive Performance

Three RCTs (75%, 3/4) evaluated the cognitive effects of MST versus ECT using different cognitive measuring tools [15,16,17]. Therefore, their data were unsuitable for meta-analysis. The results of the cognitive effects of MST versus ECT for depression are presented in Supplemental Table S2, but with inconsistent findings.

### 3.6. ADRs and Discontinuation Rate

Supplemental Table S3 summarizes the ADRs and discontinuation rate. Only two RCTs (50%, 2/4) reported ADRs, but their specific data were not described [15,16]. Only one RCT (25%, 1/4) reported discontinuation due to any reason [15].

## 4. Discussion

This updated meta-analysis studied 4 RCTs covering 86 individuals diagnosed with unipolar and bipolar depression [15,16,17,18]. The main finding of this systematic review is that the antidepressant effects of MST and ECT are equivalent, supporting the findings of two recent meta-analyses [19,37]. Three out of four (75%) RCTs investigated the impact of MST compared with that of ECT on cognitive performance for depression, but inconsistent findings were reported [15,16,17]. The included RCTs did not describe the data, detailing the rate of ADRs (four RCTs) [15,16,17,18] and discontinuation due to any reason (three RCTs), for either the MST group or the ECT group [16,17,18].

The preliminary evidence in this systematic review for the antidepressant effects of MST in subjects with depression was presented. As described in the three included RCTs [15,16,18], the response rate of MST combined with TAU in treating depression ranged from 22% to 100%, similar to the described response rate for ECT plus TAU of 22% to 70% in this systematic review. The antidepressant response rate of MST (22% to 100%) reported by the three included RCTs [15,16,18] varied in a wide range, which may be partly attributed to confounding factors, such as sample size and electrode placement. For example, only six participants were recruited in Rowny et al.’s study [18], limiting the ability to determine the actual differences of antidepressant effects, and the safety of MST against ECT. However, two large-scale noninferiority RCTs exploring MST compared with ECT for the treatment of depression are ongoing in Canada (n = 100) [47] and Brazil (n = 100) [48], which will provide a greater potential to explain the between-group differences of MST and ECT. Another confounding factor may be the electrode placement in MST. The comparator efficacy of MST on different target sites remains unclear. For patients with depression, a key target region is the dorsolateral prefrontal cortex (DLPFC), which has been implicated with the pathophysiology and treatment of MDD [49]. A recent open-label study found that MST may produce a robust antidepressant response in patients with treatment-refractory depression through transcranial magnetic stimulation and electroencephalography (TMS-EEG) of the DLPFC [50]. Thus, the optimal protocol for MST treatment for patients with MDD was unclear. Interestingly, the response rate of ECT for subjects diagnosed with schizophrenia was up to 74% [51], which was relatively higher than the response rate for MST of 37.5% to 50% [23,52]. However, another RCT found that MST had antipsychotic efficacy similar to that of ECT [53]. In recent years, MST has appeared to be an essential and interesting treatment option in depression and schizophrenia. Future RCTs with relatively larger sample sizes are needed to confirm these findings.

The mechanisms of the antidepressant effect of MST are still undetermined. Several studies found that metabolic changes in the brain might be associated with the antidepressant effect of MST [24,25,27]. For example, Hoy et al. first investigated the neural mechanism of MST’s influence, finding that MST affects regions in line with the limbic–cortical dysregulation model of depression [24]. Another study determining the impact of MST on local brain glucose metabolism found that the antidepressant effects of MST are implicated in the localized metabolic changes in regions of the brain strongly associated with depression [25]. However, whether baseline regional brain glucose metabolism levels can predict the antidepressant efficacy of MST is unclear, and further studies are needed to explore the mechanisms of the antidepressant effects of MST.

The accumulated evidence has shown that MST has less adverse cognitive effects compared to ECT [16,36]. For example, a systematic review focusing on studies investigating the cognitive effects of MST found that MST has little to no adverse cognitive effects [13]. In this systematic review, the impact of MST compared with that of ECT on cognitive functions was reported in three RCTs [15,16,17], but with inconsistent results, suggesting that the advantage of cognitive effects of MST compared with ECT remained unclear. A possible explanation for the inconsistent results was that different cognitive measuring tools were used in the three included RCTs [15,16,17], limiting our capacity to conduct meta-analysis in this study. Similarly, a recent systematic review focusing on adjunctive MST for schizophrenia reported inconsistent results regarding the impact of adjunctive MST on cognitive functions in subjects with schizophrenia [20]. Therefore, the impact of MST versus ECT on cognitive functions for depression and schizophrenia should be further investigated.

The following limitations of this systematic review should be acknowledged. First, the number of included studies (four RCTs) and the sample size (n = 86) were relatively small, precluding more robust and sophisticated analyses [15,16,17,18]. Second, the included RCTs that enrolled individuals suffering from MDD and BD rendered the study sample nonhomogeneous. Third, only three RCTs (75%, 3/4) detected cognitive effects of MST compared to ECT using different cognitive measuring tools; thus, pooling the changes of cognitive functions was not possible. Finally, this systematic review of RCTs was not registered.

## 5. Conclusions

This preliminary study suggests that MST appears to have a similar antidepressant effect as ECT for depression but with mixed findings regarding adverse cognitive effects. Further large-scale and double-blind RCTs with high-quality methods and long-term follow-up periods are required to verify the clinical findings.

## Figures and Tables

**Table 1:** Participant characteristics and MST/ECT parameters for each included study.

Study (Country)	Number of Patients ^a^	-Blinding -Analyses	Diagnosis (%)	Diagnostic Criteria	Setting	-Illness Duration ^b^ (yrs) -Sex b: Male (%)	Age ^b^: yrs (Range)	Target Site of MST	MST Device Parameters: -Device -Pulse Width (ms) -Stimulation Frequency (Hz)	ECT Device Parameters: -Device -Pulse Width (ms) -Stimulation Frequency (Hz)	Both Groups: -Anesthesia (mg/kg, Mean Dose) -Muscle Relaxant (mg/kg, Mean Dose)	-Number of Sessions (n/Week)	Jadad Score
Fitzgerald et al., 2018 (Australia)	Total: 40 MST: 21 ECT: 19	-DB -ITT	BD (16.2) and MDD (83.8)	DSM-IV	NR	-25.2 -43.2	45.9 (NR)	Left and right of the vertex	-MagVenture A/S, Denmark -NR -100	-Thymatron IV (Somatics, LLC, USA) -1.0 -NR	-Propofol (NR) -Succinylcholine (NR)	12–15 (3)	5
Kayser et al., 2011 (Germany)	Total: 20 MST: 10 ECT: 10	-OL -ITT	BD (20.0) and MDD (80.0)	DSM-IV	Inpatient	-4.8 -35.0	50.8 (NR)	Vertex	-MagVenture A/S, Denmark -0.37 -100	-Thymatron IV (Somatics, LLC, USA and Canada) -0.5 -Depending on the energy set	-Propofol (1.5–2.5, 100 mg) -Succinylcholine (1–1.5, 80 mg)	12 (2)	2
Polster et al., 2015 (Germany)	Total: 20 MST: 10 ECT: 10	-OL -ITT	MDD (100)	DSM-IV	NR	-3.6 -55.0	49.2 (18–69)	Vertex	-MagVenture A/S, Denmark -NR -100	-Thymatron IV (Somatics, LLC, USA and Canada) -0.5 -Depending on the energy set	-Propofol (1.5, 100 mg) -Succinylcholine (1, 70 mg)	10–12 (2)	1
Rowny et al., 2020 (USA)	Total: 6 MST: 3 ECT: 3	-NR -ITT	BD (NR) and MDD (NR)	NR	NR	-NR -NR	64.8 (57–74)	Bilaterally at F5–F6 ^c^	-MagVenture A/S, Denmark -0.37 -50	-Thymatron -0.25 -NR	-NR -NR	8–18 (3)	1

^a^ Data were extracted based on random assignment. ^b^ Available data were extracted based on the mean baseline value of each included trial. ^c^ The target site was decided according to the International 10–20 EEG system. Abbreviations: BD = bipolar disorder; DB = double blind; DSM-IV = Diagnostic and Statistical Manual of Mental Disorders 4th edition; ECT = electroconvulsive therapy; ITT = intent-to-treat; MDD = major depressive disorder; MST = magnetic seizure therapy; NR = not reported; OL = open label; yrs = years.

**Table 2:** GRADE analyses: MST versus ECT for depression.

Primary and *Secondary* Outcomes	Study (Subjects)	Risk of Bias	Inconsistency	Indirectness ^b^	Imprecision	Publication Bias	Large Effect	Overall Quality of Evidence ^a^
Study-defined response	3 (63)	No	No	No	Serious ^d^	None detected	No	+/+/+/−/; Moderate
Study-defined remission	3 (63)	No	No	No	Serious ^d^	None detected	No	+/+/+/−/; Moderate
HAMD total scores	3 (63)	No	No	No	Serious ^c^	None detected	No	+/+/+/−/; Moderate

Abbreviations: ECT = electroconvulsive therapy; GRADE = grading of recommendations assessment, development, and evaluation; HAMD = Hamilton Depression Scale; MST = magnetic seizure therapy. ^a^ GRADE Working Group grades of evidence: high quality = further research is very unlikely to change our confidence in the estimate of effect; moderate quality = further research is likely to have an important impact on our confidence in the estimate of effect and could change the estimate; low quality = further research is very likely to have an important impact on our confidence in the estimate of effect and is likely to change the estimate; very low quality = we are very uncertain about the estimate. ^b^ Meta-analytic results presented a serious inconsistency when I^2^ values were greater than 50% or *p* < 0.1 in the *Q* statistics. ^c^ For continuous outcomes, N < 400. ^d^ For dichotomous outcomes, N < 300.

## Data Availability

The data are available from the corresponding author on request.

## Supplementary Materials

The following supporting information can be downloaded at: https://www.mdpi.com/article/10.3390/jpm13030449/s1, Figure S1: Risk of bias; Table S1: MST versus ECT for depression: clinical effects; Table S2: MST versus ECT for depression: cognitive functions; Table S3: MST versus ECT for depression: ADRs and discontinuation rates.
