# Supplementary material for: Comparison of Efficacy and Safety of Magnetic Seizure Therapy and Electroconvulsive Therapy for Depression: A Systematic Review
Source: J Pers Med. 2023 Feb 28;13(3):449. doi: 10.3390/jpm13030449 (PMC10057006; doi:10.3390/jpm13030449)
Supplement: Supplementary file 1 [file jpm-13-00449-s001.zip › jpm-2216356-supplementary.pdf]

**Supplemental Table S1.** MST versus ECT for depression: clinical effects

| Study | Clinical effects | MST group at endpoint (mean ± SD) | ECT group at endpoint (mean ± SD) | Findings[a] |
|---|---|---|---|---|
| Fitzgerald et al., 2018 | IDS | 38.1±14.8 | 35.3±15.4 | NR |
| | QIDS | 17.3±7.4 | 18.6±9.0 | NR |
| Kayser et al., 2011 | MADRS | 15.9±9.7 | 16.1±9.5 | *P*>0.05 |
| | BDI | 25.8±17.1 | 24.5±15.6 | *P*>0.05 |
| Polster et al., 2015 | - | - | - | - |
| Rowny et al., 2020 | - | - | - | - |

[a]ANOVA for repeated measures with the factor treatment (MST versus ECT) as between subject variable.

Abbreviations: ANOVA=analyses of variance; BDI=Beck Depression Inventory; ECT=electroconvulsive therapy; IDS=Inventory of Depressive Symptomatology; MADRS=Montgomery-Asberg Depression Scale; MST=magnetic seizure therapy; NR=not reported; QIDS=Quick Inventory of Depressive Symptomatology Shortened Patient version

**Supplemental Table S2.** MST versus ECT for depression: cognitive functions

| Study | Cognitive functions | | | |
|---|---|---|---|---|
| | **Cognitive domain** | **MST group at endpoint (mean ± SD)** | **ECT group at endpoint (mean ± SD)** | **Findings[a]** |
| Fitzgerald et al., 2018 | AMI | 21.8±3.7 | 21.2±4.7 | NS[b] |
| | BVMT total | 23.2±7.9 | 19.7±7.7 | NS[b] |
| | BVMT Delayed recall | 9.0±3.3 | 6.9±4.1 | NS[b] |
| | BVMT recognition | 11.4±1.4 | 10.8±2.1 | NS[b] |
| | RAVLT total score | 48.9±11.3 | 44.7±12.5 | NS[b] |
| | RAVLT recognition list total | 35.1±5.0 | 31.9±6.9 | NS[b] |
| | Story Memory 1 | 11.8±3.8 | 9.9±4.5 | NS[b] |
| | Story Memory 2 | 8.9±3.6 | 7.3±4.0 | NS[b] |
| | Story Memory delayed recall 1 | 9.7±3.6 | 6.9±4.6 | NS[b] |
| | Story Memory delayed recall 2 | 7.3±3.6 | 4.4±3.7 | NS[b] |
| | Digit span forward | 11.9±2.7 | 10.3±2.3 | NS[b] |
| | Digit span backward | 8.2±2.6 | 5.9±2.4 | NS[b] |
| | REY total copy score | 28.8±3.1 | 31.3±2.6 | NS[b] |
| | Digit symbol coding | 63.6±15.6 | 52.6±17.2 | ***P*=0.009** |
| | Trail Making, test A | 26.2±9.7 | 34.0±21.9 | NS[b] |
| | Trail Making, test B | 64.1±29.1 | 99.5±58.0 | NS[b] |
| | Verbal fluency total | 42.1±9.7 | 34.1±11.2 | NS[b] |
| | Stroop colour word | 66.6±14.1 | 57.9±10.8 | NS[b] |
| | Verbal paired associates: easy | 17.0±1.2 | 16.4±1.5 | NS[b] |

| | | | | | |
|---|---|---|---|---|---|
| | Verbal paired associates: hard | | 7.9±2.9 | 6.3±4.2 | NS[b] |
| Kayser et al., 2011 | Verbal learning and memory: words | WORDS immediate recall | 6.9±1.4 | 6.4±2.2 | NS |
| | | WORDS immediate recognition | 13.3±1.8 | 13.2±2.4 | NS |
| | | WORDS delayed recall | 2.7±2.1 | 1.7±2.0 | NS |
| | | WORDS delayed recognition | 12.1±1.4 | 12.3±2.3 | NS |
| | Visual spatial learning and memory: shapes | SHAPES immediate recognition | 6.5±1.5 | 4.6±2.4 | NS |
| | | SHAPES delayed recognition | 4.7±1.9 | 3.5±2.2 | NS |
| | Verbal memory: Wechsler Memory Scale | Logical memory I: immediate recall | 14.3±4.1 | 13.8±6.1 | NS |
| | | Logical memory II: delayed recall | 12.9±5.5 | 10.3±5.8 | NS |
| | Abstract questions | | 4.5±0.9 | 3.8±1.4 | NS |
| | Picture test | | 4.8±0.6 | 4.7±0.7 | NS |
| | Verbal fluency | Semantic categorial | 26.5±8.5 | 22.2±8.3 | NS |
| | | Formal lexical | 15.1±7.4 | 11.2±4.7 | NS |
| | Neglect | Geometric forms | 52.0±10.5 | 69.5±20.0 | ***P*<0.05** |
| | | Letters | 63.6±13.5 | 72.5±22.4 | NS |
| | | Nongeometric forms | 87.9±15.1 | 84.9±30.7 | NS |
| Polster et al., 2015 | Immediate recall | | 20.4 ±6.2 | 14.3 ± 5.8 | ***P*<0.05** |
| | Delayed recall | | 51.1% ± 21.8% | 23.2% ± 24.6% | **P=0.015** |
| | Cued recall | | - | - | NS |
| Rowny et al., 2020 | - | - | - | - | - |

Bolded values are *P*<0.05.

[a]The differences between the two treatment groups were assessed with independent samples *t* tests.

[b]For these analyses, the results were only considered significant if the *P* value was <0.01 for the included study.

Abbreviations: AMI=Autobiographical Memory Interview; BVMT=Brief Visual Spatial Memory Test; ECT=electroconvulsive therapy; MST=magnetic seizure therapy; NR=not reported; RAVLT=Rey Verbal Auditory Learning Test; REY=Rey Complex Figure Test (copy); NS=not significant

**Supplemental Table S3.** MST versus ECT for depression: ADRs and discontinuation rates

| Study | ADRs | | | | Discontinuation due to any reason | | |
|---|---|---|---|---|---|---|---|
| | Events | Total (%) | MST group (%) | ECT group (%) | Total (%) | MST group (%) | ECT group (%) |
| Fitzgerald et al., 2018 | Awareness prior to recovery from muscle paralysis | NR | NR | 0 | 3 (7.5) | 3 (14.3) | 0 (0) |
| Kayser et al., 2011 | Headache | NR | 0 | NR | NR | | |
| | Muscle pain | NR | 0 | NR | | | |
| | Nausea | NR | 0 | NR | | | |
| Polster et al., 2015 | NR | NR | | | NR | | |
| Rowny et al., 2020 | NR | NR | | | NR | | |

Abbreviations: ADRs=adverse drug reactions; ECT=electroconvulsive therapy; MST=magnetic seizure therapy; NR=not reported

**Supplemental Figure S1:** Risk of bias

| | *Random sequence generation (selection bias)* | *Allocation concealment (selection bias)* | *Blinding of participants and personnel* | *Blinding of outcome assessment (Symptom reduction, response)* | *Incomplete outcome data addressed (attrition bias)* | *Selective reporting (reporting bias)* | *Other sources of bias* |
|---|---|---|---|---|---|---|---|
| Fitzgerald et al., 2018 | + | ? | + | + | + | + | ? |
| Kayser et al., 2011 | ? | ? | - | - | + | + | ? |
| Polster et al., 2015 | ? | ? | - | - | + | ? | ? |
| Rowny et al., 2020 | ? | ? | ? | ? | ? | ? | ? |

+: Low risk of bias, -: High risk of bias,?: Unclear risk of bias
